# Means of Avoiding the Electric Current during a Thunder-Storm

**Published:** 1844-09-21

**Authors:** 


MEANS OF AVOIDING THE ELECTRIC CURRENT DURING A THUNDER-STORM.

The place of greatest security is a railway carriage at a distance from the engine. The rails dissipate the negative charge. An individual is also perfectly protected in an iron vessel at sea. In a house the most secure position is the most central point, if in an apartment without a flue the better. A cellar affords good security against the upward or negative current, whilst a bed affords the best security against the effects of the downward or positive current. To avoid danger, if an individual is on high ground, he should descend; if in a field, he should retire to a hedge; within twenty yards of a tree is a safe position, and, if possible, lying down. It is better not to remain on horseback during a thunder-storm, and those who are in carriages should keep the windows closed. At sea it is necessary to avoid the masts and forecastle of the vessel and to retire to a hammock. If in a boat, rear a wet oar near its head, retire to the stern and lie down. A position near a lake or stream of water is highly dangerous—*Turley on Thunder-storms: Medico. Chir. Review.*

